# Indication of elective contralateral neck dissection in squamous cell carcinoma of the hypopharynx

**DOI:** 10.1016/S1808-8694(15)30485-7

**Published:** 2015-10-19

**Authors:** Ali Amar, Rogério Aparecido Dedivitis, Abrão Rapoport, André Luiz Quarteiro

**Affiliations:** 1 Doctor in medicine, post-graduate course in otorhinolaryngology and head & neck surgery, Sao Paulo Federal University, Sao Paulo. Assistant physicians in the otorhinolaryngology and head & neck surgery unit, Heliopolis Hospital, Sao Paulo; 2 Doctor in medicine, post-graduation course in otorhinolaryngology and head & neck surgery, UNIFESP (Escola Paulista de Medicina). Physician; 3 Habilitation (livre docente) professor, surgery department of the Sao Paulo university medical school, São Paulo. Coordinator of the post-graduate course in otorhinolaryngology and head & neck surgery, Heliopolis Hospital, Sao Paulo; 4 Specialist in otorhinolaryngology; post-graduate student of the health sciences, Heliopolis Hospital, Sao Paulo. Post-graduate course in health sciences, Heliopolis Hospital, Sao Paulo

**Keywords:** squamous cell carcinoma, neck dissection, hypopharynx, lymphatic metastasis

## Abstract

Lymph node metastases (LNM) are common in hypophariyngeal carcinomas; the neck dissection is an important therapeutic approach.

**Aim:**

to analyze the incidence and distribution of LNM and failures in treating the contralateral neck.

**Methods:**

a retrospective study of 174 patients with hypopharyngeal cancer treated from 1978 to 2003. The distribution of LNM and regional recurrences were evaluated.

**Results:**

44% of the cases were false negatives and 4.9% were false positives. Among the 48 patients who underwent bilateral ND, 29 had bilateral metastases and one had contralateral metastasis. Contralateral neck recurrences occurred in 12 cases that underwent unilateral ND. Among the nine patients with contralateral neck recurrence alone, eight were surgically salvaged. The risk of contralateral metastases was related to clinical staging (p=0.003) and involvement of the medial wall of the pyriform sinus (p=0.03), but not to radiotherapy (p=0.28).

**Conclusion:**

Contralateral metastases were more frequent when the medial wall of the pyriform sinus was affected, in the presence of ipsilateral palpable metastases and clinical stage IV.

## INTRODUCTION

Lymph node metastases occur frequently in hypopharyngeal carcinomas, and may be a patient's first manifestation. The rate of false negatives is about 40% even in cases with no palpable lymph nodes. Therapeutic or elective neck dissection is an important aspect of therapy.^1^ Knowledge about regional dissemination is relevant for unilateral or bilateral neck dissection, especially when done electively, since the extent of this procedure will reflect on postoperative morbidity and mortality and regional disease control. Although lymphatic drainage and the distribution of metastases are known, indications for neck dissection are still debated.

The purpose of this study was to evaluate the incidence and distribution of lymph node metastases and treatment failure of regional disease in hypopharyngeal squamous cell carcinoma, with an emphasis on the treatment of the contralateral neck.

## METHOD

The Research Ethics Committee of the institution in which research was carried out approved this study (number 497).

The files of hypopharyngeal squamous cell carcinoma patients undergoing neck dissection were reviewed. Patients treated previously, those with multiple synchronic tumors, or those with no description of the lymph nodes involved were excluded from the sample. The sample consisted of 174 cases treated from January 1978 to December 2003. The location of metastases was stratified into ten levels and regrouped according to the American Head and Neck Society (AHNS) standards.^2^ Based on the description of the initial physical examination and surgical findings, the incidence, laterality and relation with the primary tumor of lymph node metastases was defined at these levels. The TNM-2000 (UICC-AJCC) classification system was used for staging all patients. Assessment of regional recurrence in the non-dissected side was done before applying postoperative radiotherapy.

Statistical analysis consisted of the chi-square test with the Yates correction and the difference between two proportions test; bicaudal p values below 0.05 were accepted. Disease-free survival and cervical control were calculated using the Kaplan-Meier actuarial method.

In 174 patients, 222 neck dissections were undertaken; there were 206 radical neck dissections and 16 selective neck dissections. There were 163 male and 11 female patients. The mean age was 56 years, ranging from 36 to 80 years. The epicenter of lesions was the pyriform recess in 171 patients, and the posterior wall in 3 patients. Two patients had stage I disease, four patients had stage II disease, 46 patients had stage III disease, and 122 patients had stage IV disease (Table 1). Mean follow-up was 31 months after therapy.

**Table 1:** Distribution of patients according to T and N staging.

T	N0	N1	N2a	N2b	N2c	N3	Nx	Total
1	2	0	0	0	0	0	0	2
2	4	6	4	2	1	4	0	21
3	20	20	21	22	11	15	0	109
4	8	6	5	4	9	9	1	42
Total	34	32	30	28	21	28	1	174

## RESULTS

Of 34 N0 patients, 15 (44%) had histological metastases; one had bilateral metastases. Of 140 N+ patients, seven (4.9%) were false positive. Of 48 patients undergoing bilateral neck dissection, 29 had bilateral metastases and one patient had exclusively contralateral metastases confirmed histologically; of these 29 patients, 22 had a clinical diagnosis of bilateral or contralateral metastases.

**Figure 1:**
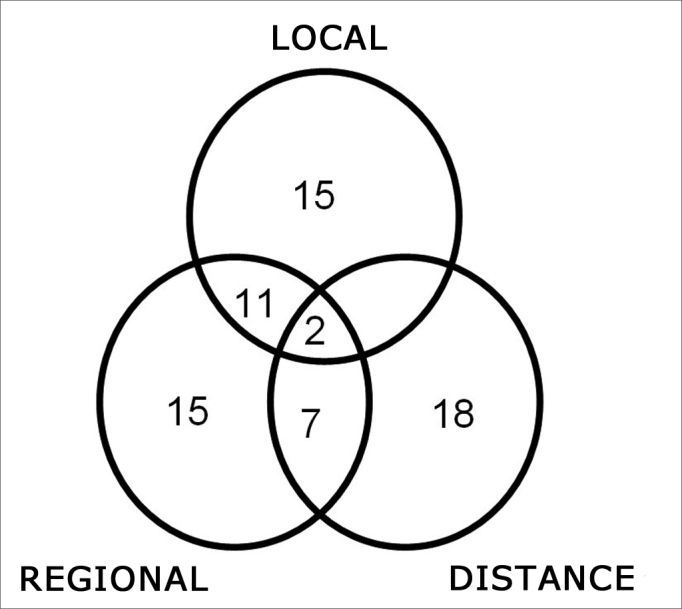
Recurrence of hypopharyngeal squamous cell carcinoma.

Locoregional recurrences were diagnosed in 50 cases (Fig. 1). Distance metastases occurred in 27 cases. Six peritracheostomy, one retropharyngeal and 14 second primary tumors were also identified.

**Figure 2:**
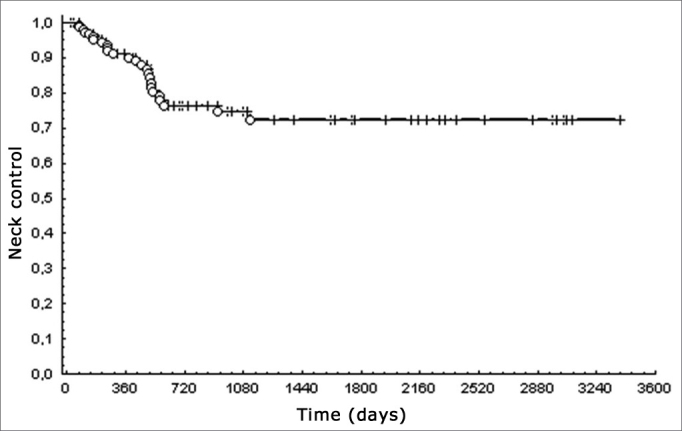
Cervical control in hypopharyngeal squamous cell carcinoma.

Cervical control was 72%; overall 5-year survival was 28% (Fig. 2).

Contralateral cervical recurrences were found in 12 cases undergoing unilateral neck dissection; three of these had concomitant pharyngeal recurrences. Of nine patients with single contralateral cervical recurrences, one patient had non-resectable disease and eight patients underwent surgical salvage with or with no postoperative radiotherapy. Of eight salvaged patients, three had recurrences in other sites and one recurred regionally. In the four remaining patients, one was lost to follow-up soon after treatment and the remaining three had controlled disease at 6, 19 and 162 months.

There were four (5%) contralateral recurrences in 79 cases treated with radiotherapy cases following unilateral neck dissection, and five (10.6%) recurrences in 47 patients treated only surgically (p=0.28). Adding these recurrences with the metastases found on histological exams results in 39 patients (22%) with contralateral metastases, of which 22 cases (56%) were found in the physical examination. Table 2, Table 3 show the distribution of ipsilateral and contralateral metastases.

**Table 2:** Distribution of histological metastases (pN) in contralateral neck dissection in cervical recurrences of non-operated necks.

	Elective n=21	Therapeutic n=27	Recurrence n=9
Level			
I	1	1	–
IIa	4	15	5
IIb	–	5	–
III	3	12	3
IV	–	10	1
V	–	6	–
VI	–	–	–

**Table 3:** Distribution of ipsilateral histological metastases (pN).

	N0 n=34	N+ n=140	Total n=174
Level			
I	–	6	6
IIa	9	100	109
IIb	1	30	31
III	5	76	81
IV	4	43	47
V	–	32	32
VI	1	8	9

The medial wall of the pyriform sinus was involved in 125 of 174 cases; 26 of 29 patients with bilateral metastases had tumors on this site (p=0.03). Bilateral metastases occurred in 10 cases (23%) of 43 tumors that crossed the midline, and in 20 cases (15%) of 131 unilateral tumors (p=0.25).

Table 4 shows that the risk of contralateral metastases was related with staging (p=0.003). Of nine patients that presented contralateral metastases, six had stage IV disease and three had stage III disease.

**Table 4:** Incidence of bilateral histological metastases according to the clinical stage (p=0.003)

		Bilateral metastases	
Stage	Yes	No	Total
I e II	0 (0%)	6 (100%)	6
III	1 (2%)	45 (97%)	46
IV	28 (22%)	94 (77%)	122
Total	29 (16%)	145 (83%)	174

## DISCUSSION

Hypopharyngeal lymphatic drainage and lymph nodes involved in disease are well known.^3^, ^4^, ^5^ False positive cases are rare; metastases may occur at nearly all lymph node levels, although the frequency is low in level I. Contralateral metastases are distributed similarly to the ipsilateral size, both on selective and therapeutic neck dissection. Level I metastases were seen in seven cases. This incidence means that level I may be saved even in therapeutic neck dissection, since recurrences in this site may be salvaged.

Lesions involving the medial wall of the pyriform recess – even when not crossing the midline – are at a higher risk of presenting contralateral metastases; this is due to crossed lymphatic drainage in this area.^6^,^7^ Kowalski et al. identified the hemilaryngeal fixation and epilaryngeal or posterior wall involvement as risk factors for contralateral metastases.^8^ Although both sides of the neck are exposed in surgery, the incidence of metastases in N0 cases does not justify elective contralateral neck dissection; this procedure is done only in retrocrichoid or posterior wall lesions (midline).^1^, ^7^, ^9^, ^10^ A high rate of contralateral metastases occurs only cases with clinical stage IV and when ipsilateral lymph nodes are present; in these cases, elective neck dissection is justified.

There was no significant statistical difference in the recurrence rate in the non-dissected neck after radiotherapy of both sides. Radiotherapy, however, is an option if there is subclinical disease in the contralateral neck, since most patients have additional criteria for postoperative radiotherapy. Hypopharyngeal tumors usually present at advanced stages and recurrence in the operated area is difficult to salvage; locoregional control is more effective with combined therapy.^1^,^8^ Opting for radiotherapy in elective treatment of the contralateral neck requires adequate patient follow-up and prompt salvage therapy.

Level VI metastases were seen in 5% of patients; this rate rises to 8% in peritracheostomy recurrences are taken into account. Other authors have reported a 0% to 20% rate at this level.^11^,^12^ Level VI metastases occur more often in tumors of the larynx and cervical esophagus; recurrences in these areas, however, can rarely be salvaged successfully, and routine neck dissection is indicated in such cases.^11^ Retropharyngeal lymph nodes were not evaluated in this series. Computed tomography was not available for most patients, and the routine at this unit consisted of assessing patients intraoperatively and removing only palpable lymph nodes; there was no systematic report on these lymph nodes. Metastases were found in 17% of cases in patients undergoing routine neck dissection of this area; however, systematic removal did not alter survival rates.^13^

Contralateral metastases are a possible cause of failure in the treatment of hypopharyngeal tumors. The incidence may have been underestimated, since many patients were treated postoperatively with radiotherapy, and lymph nodes were assessed based on few histological sections; the rate, however, was similar to other published results.^8^,^14^ Although contralateral metastases were diagnosed in 22% of patients, low risk subgroups may be identified. Thus, in many cases, elective contralateral neck dissection could be avoided in cases with tumors of the pyriform recess, which would decrease morbidity and postoperative complications. Malnutrition and comorbidity are common findings in patients hypopharyngeal carcinomas; the risk-benefit ratio of elective contralateral neck dissection cannot be established in a retrospective study. Regional control may be attained in most patients, in spite of an unfavorable prognosis when there are lymph node metastases; in these cases overall survival remains low.^1^,^8^

## CONCLUSION

Contralateral metastases were more frequent in hypopharyngeal carcinomas when the medial wall of the pyriform recess was involved, with ipsilateral palpable metastases and clinical stage IV.
